# Tumor-related epilepsy and post-surgical outcomes: tertiary hospital experience in Vietnam

**DOI:** 10.1038/s41598-023-38049-1

**Published:** 2023-07-05

**Authors:** Viet-Thang Le, Anh Minh Nguyen, Tuan Anh Pham, Phuc Long Nguyen

**Affiliations:** 1 Faculty of Medicine, University of Medicine and Pharmacy, 217 Hong Bang Street, 11th Ward, 5th District, Ho Chi Minh City, 700000 Vietnam; 2 Department of Neurosurgery, University Medical Center, UMC, 215 Hong Bang Street, 11th Ward, 5th District, Ho Chi Minh City, 700000 Vietnam; 3 Department of Neurosurgery, Nguyen Tri Phuong Hospital, 468 Nguyen Trai Street, 8th Ward, 5th District, Ho Chi Minh City, 700000 Vietnam

**Keywords:** CNS cancer, Epilepsy

## Abstract

Seizures have a significant impact on the quality of life of those who suffer. This study aimed to evaluate the variables that influence the incidence of seizures during the perioperative period and effective measures to enhance epilepsy outcomes among individuals undergoing surgical resection of brain tumors. The authors carried out a prospective observational analysis of all patients who experienced seizures before their brain tumor surgery at UMC, HCMC between 2020 and 2022. 54 cases presented with seizures were enrolled for the study, generalized seizure was the most prevalent seizure type (61.1%), followed by focal seizure (29.6%). The majority of patients presented with seizures are those who were diagnosed with glioma. Low-grade gliomas and frontotemporal lobe tumors increase the postoperative risk of seizure. Other predictive factors are a prolonged history of seizure, especially resistant epilepsy and major peritumoral edema. In contrast, gross total resection reduces postoperative seizure incidence. There was correlation between Ki67 proliferation index and seizure incidence in both low-grade and high-grade gliomas. ECoG made insubstantial difference in enhancing the epilepsy surgery outcome. Overall, 88.9% of patients were seizure-free at 6 months of follow-up (Engel Class I), 7.4% were almost seizure-free (Class II), and 3.7% had significant improvement (Class III), figures for 12-month follow-up were 87.0%, 9.3%, and 3.7% respectively. A shorter history of seizure and gross-total resection appear to be associated with a favorable prognosis for seizure control.

## Introduction

Epilepsy is one of the most common neurological diseases, affecting millions of people worldwide. Various mechanisms result in epilepsy, and brain tumors are the most frequent cause. The risk of developing epilepsy in patients with brain tumors depends on distinct factors. Epilepsy occur in 60–80% of gliomas. Low-grade gliomas are associated with a high seizure incidence (85–92%). In contrast, glioblastomas and meningiomas generate epilepsy activities at a lower rate (20–50%). Tumor location is also suggested to be involved in seizure establishment. Lesions in the temporal, frontal, and parietal regions produce a higher seizure frequency than other brain regions. Overall, 70% of seizures can be effectively controlled with anti-seizure medications (ASMs). However, in addition to ASMs, treatment of brain tumor-related epilepsy requires the involvement of other management. The management aims to simultaneously eliminate the seizures and remove the tumor. In light of advanced neurosurgical techniques and equipment, targeting factors related to the epileptogenicity of patients with brain tumors is crucial to enhance the effectiveness and safety of epilepsy treatment and achieve treatment goals. This study investigates predictors and measurements affecting seizure outcomes in the first year after surgery of different types of epileptogenic tumors.

## Results

Table 1 described all clinical features of the total 54 included patients. The sex ratio was close to 1:1. The mean age was 41.7 ± 15.6 and ranged from 18 to 75 years old. There were 53.7% of patients admitted to the hospital because of an onset seizure, and the rest 46.3% of patients had a previous history of epilepsy, in which refractory epilepsy accounted for 9.3% (5 cases). 31.5% of patients had epilepsy more than 1 year before the surgery, and 11.7% of those were refractory epilepsy. A prolonged history of epilepsy neither had an impact on preoperative uncontrolled seizure nor postoperative seizure. However, at 6 and 12 months after surgery, those who suffered from > 1 year of epilepsy history had a higher risk of seizures recurrence (p = 0.009 and p = 0.003 respectively). Generalized seizures were the most prevalent seizure type (61.1%), followed by focal seizures (29.6%). 70.4% of patients experienced motor onset, while 13.0% of those observed did not have motor manifestation. The preoperative seizure type was also associated with postoperative outcomes, patients with simple focal seizures often fail to achieve meaningful epilepsy regression after surgery. Seizure occurred during postoperative period was the predictor for inadequate seizure control at follow-ups. Factors affect seizure outcome after surgery and at follow-ups was demonstrated in Table 2.

**Table 1:** Clinical features of patients before and after surgery.

Characteristic	N (%)
Gender	
Female	26 (48.1%)
Male	28 (51.9%)
Age (mean ± SD, range: )	41.7 ± 15.6
Duration of epilepsy	
< 1 year	37 (68.5%)
> 1 year	17 (31.5%)
Refractory epilepsy	5 (9.3%)
Pre-op GCS (mean ± SD)	14.9 ± 0.2
Post-op GCS (mean ± SD)	14.9 ± 0.3
Location	
Temporal	19 (35.2%)
Frontal	12 (22.2%)
Parietal	9 (16.7%)
Occipital	6 (11.1%)
Insular	2 (3.7%)
Infratentorial	1 (1.9%)
> 2 lobes	5 (9.3)
Volume cm^3^ (mean ± SD)	40.1 ± 53.5
Peritumoral edema	
Minor	21 (61.1%)
Major	33 (38.9%)
Pathology	
LGG	9 (16.7%)
HGG	22 (40.7%)
Meningioma	15 (27.8%)
Others	8 (14.8%)
Removal extension	
Subtotal resection	13 (24.1%)
Grostotal resection	35 (64.8%)
Extra-lesional resection	6 (11.1%)
Chemotherapy	19 (35.2%)
Radiotherapy	11 (20.4%)
KPS	
> 70	39 (72.2%)
< 70	15 (27.8%)

**Table 2:** Factors affect seizure outcome after surgery and at follow-ups.

	Post-operative			6 months			12 months		
	Seizure (−)	Seizure (+)	p value	Engel I	Engel II–IV	p value	Engel I	Engel II–IV	p value
Duration from onset > 1 year	13	4	0.243	12	5	*0.009*	11	6	*0.003*
Refractory epilepsy	2	3	*0.019*	3	2	0.089	3	2	0.120
Focal seizure	11	5	*0.041*	12	4	0.056	10	6	*0.002*
Peritumoral edema: major	15	6	*0.045*	15	6	*0.002*	14	7	*0.001*
Low grade glioma	5	4	*0.02*	4	5	*0.002*	5	4	*0.011*
Frontotemporal lobe	24	7	*0.010*	26	5	*0.034*	25	6	*0.028*
Post op seizure	–	–	–	4	4	*0.003*	4	4	*0.006*
GTR	32	3	0.113	35	0	*0.001*	34	1	*0.006*
ELR	4	2	0.213	4	2	0.127	5	1	1.000
ECoG guidance	2	2	0.100	2	2	0.057	2	2	0.077
Chemotherapy	–	–	–	18	1	0.408	17	2	1.000
Radiotherapy	–	–	–	10	1	1.000	10	1	1.000

GTR: gross-total resection, ELR: extra-lesional resection, ECoG: electrocorticography.

Significant values are in italics.

**Figure 1:**
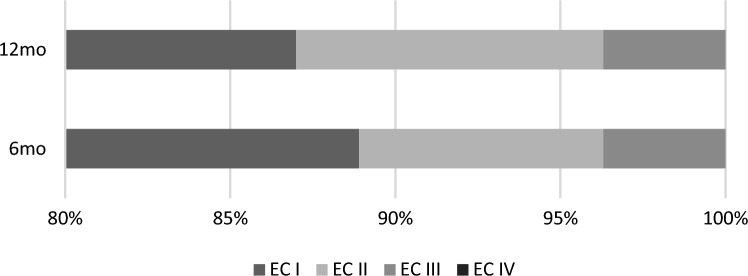
Seizure outcome at 6 and 12 months (EC: Engel Class). There was no one experienced a disabling seizure (Engel Class IV).

Overall, 88.9% of patients were seizure-free at 6 months of follow-up (Class I), 7.4% were almost seizure-free (Class II), and 3.7% had significant improvement (Class III). These figures for 12-month follow-up were 87.0%, 9.3%, and 3.7% respectively. There was no one experienced a disabling seizure (Class IV) (Fig. 1).

**Figure 2:**
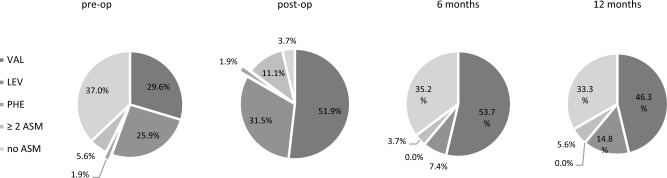
Preoperative and postoperative ASM usage. Before surgery, 63% of the patients were prescribed ASM, with valproate and levetiracetam were the most frequently used agent. Resistant epilepsy requires multiple ASM, of which Levetiracetam and Valproate were the most common combination. Almost all patients were prescribed ASM postoperatively, and ASM was gradually tapered over the follow-up time. At 12 months of follow-up, 33.3% of patients achieved complete ASM withdrawal.

Before surgery, 63% of the patients were prescribed ASM, with valproate was the most frequently used agent (29.6%), followed by levetiracetam (25.9%). Resistant epilepsy requires multiple ASMs, of which Levetiracetam and Valproate were the most common combination. We noticed almost all patients were prescribed ASM postoperatively, albeit ASM was gradually tapered over the follow-up time. At 12 months of follow-up, 33.3% of patients achieved complete ASM withdrawal in our study (Fig. 2).

In our population, glioma was the most prevalent pathology (57.4% of cases), of which HGG accounted for 71.0%. 4 out of 5 patients with refractory epilepsy were later found to have glioma diagnosis, but pathologies did not statistically relevant to preoperative uncontrolled seizure (p = 0.244). Instead, low grade gliomas were linked to postoperative seizure and Engel class II-IV epilepsy outcome at 6 and 12 months of follow-up (p = 0.02, p = 0.0002, and p = 0.011 in that order). The mean tumor size was 40.1 cm^3^, ranging from 1.4 to 288.8 cm^3^. However, tumor size was not associated with the rate of uncontrolled epilepsy. Major peritumoral edema was present in 38.9% of patients and was mostly due to HGG (61.9%) while one-third of LGG accompanied with major peritumoral edema. Most meningiomas did not cause edema except for significantly large tumors.

**Figure 3:**
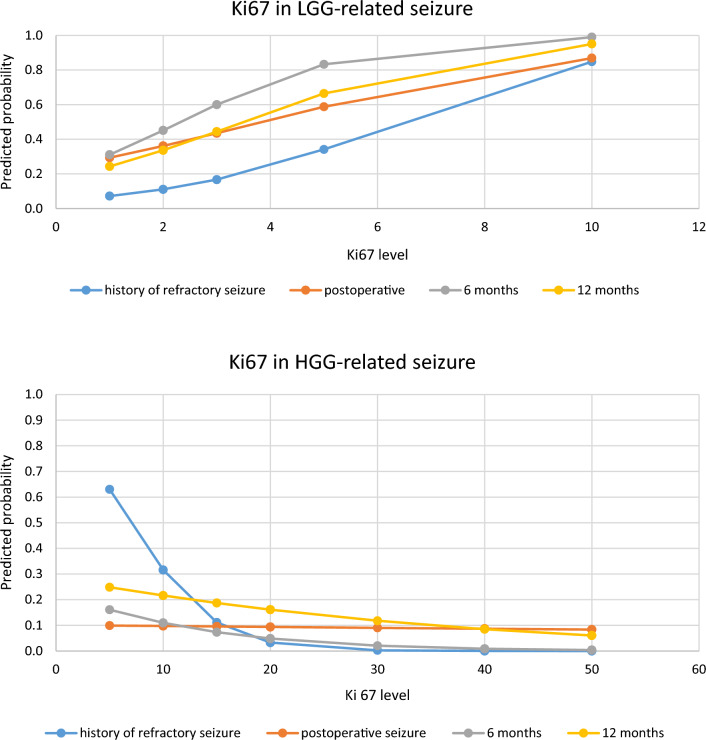
Predicted probability of developing LGG- and HGG-related seizure by Ki67. Upper chart: there was a positive correlation between Ki67 level in LGG and preoperative refractory epilepsy, postoperative, 6-month and 12-month seizure rate. Lower chart: among HGG, high Ki67 proliferation index was associated with a lower incidence of both preoperative uncontrolled epilepsy and postoperative seizure.

We performed immunohistochemistry studies for all glioma pathologies. All samples are positive with Glial fibrillary acidic protein (GFAP). HGG produced a significantly high Ki67 proliferation index (5–50%), in comparison with just 1–10% in LGG. In logistic regression analysis, Fig. 3 demonstrates a positive correlation between Ki67 level in LGG and preoperative refractory epilepsy, postoperative, 6-month and 12-month seizure rate (OR = 1.609, OR = 1.36, OR = 1.82, and OR = 1.58 respectively). In contrast, considering HGG, high Ki67 proliferation index was associated with a lower incidence of both preoperative uncontrolled epilepsy and postoperative seizure (OR = 0.77, OR = 0.99, OR = 0.92, OR = 0.96, in that order).

The degree of cerebral edema was not associated with the incidence of uncontrolled preoperative seizures (p = 0.069). Tumors were most common in the temporal lobe (42.6%), followed by the frontal lobe (27.8%) and parietal lobe (13.0%). Though most refractory epilepsy cases had tumors in the temporal lobe, we did not find a correlation between tumor location and the history of resistant epilepsy (p = 0.086). However, tumors located at the temporal and frontal lobes increased the rate of the postoperative seizure (p = 0.010), 6 months seizure (EC Class II–IV) (p = 0.034), and 12 months seizure (p = 0.028). Regarding the extent of tumor removal, most surgeries were gross-total removal (Meningiomas 100%, LGG 55.6%, and HGG 59.1%). There were 4/9 cases of LGG and 2/22 cases of HGG underwent extra-lesional resection (ELR). We performed ELR mainly for small tumors located in non-functional areas. ECoG was used to guide the surgery to remove the entire epileptic foci and preserve the functional outcome. GTR was associated with good postoperative epilepsy remission and during follow-up (Engel Class I), whereas ELR did not have a significant enhance on the outcome. Major peritumoral edema, prolonged duration from the onset (> 1 year), focal seizure type, and uncontrolled preoperative seizure were related to poor seizure control at follow-up. These variables were entered into multivariate analysis using logistic regression. However, none of these was an independent predictor for seizure control at 6 and 12 months.

## Discussion

**Figure 4:**
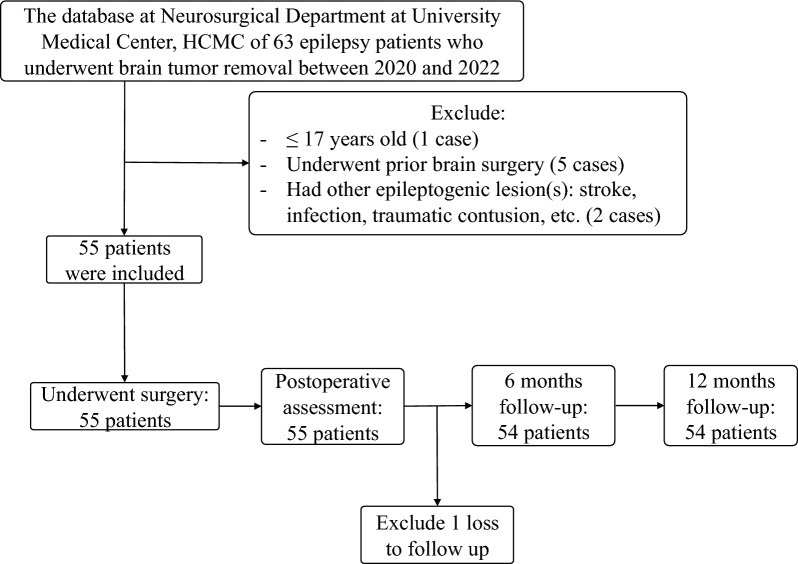
Patient selection flow diagram for final assessment.

Brain tumors have a very variable frequency of seizure activity. Although many forms of brain tumors can cause epilepsy, patients with low-grade intrinsic lesions are more likely to experience seizures. Our study did not enroll all brain-tumor patients to investigate (Fig. 4). Nevertheless, up to 90% of patients with low-grade gliomas have been observed to have epilepsy^1,2^. High-grade gliomas also produce seizure symptoms, however, seizure rate is commonly less frequent compared to low-grade lesions^3–5^.

Other low-grade tumors, such as gangliogliomas and dysembryoplastic neuroepithelial tumors (DNETs), are also reported to commonly cause seizures^6^. This could be explained by several mechanisms, in which molecular genetics play an important role. Chen and Zhong shared similar evidence that tumor grade and location may not be as relevant to epileptogenesis as tumor molecular genetic markers. Seizures are more frequent in gliomas that carry an isocitrate dehydrogenase-1 (IDH1) mutation or p53 overexpression (> 40%)^5,7^. It is usually accepted that glioma-related seizures physiologically result in vascular compression, structural displacement of the surrounding cortex, ischemia, and infarcted hemorrhage. These injuries activate local inflammation, hypoxic-ischemic damage, and damage to the blood–brain barrier, which predisposes to epileptogenesis^8^.

Prior studies have noted the relationship between cortical location and seizures^9^. Individuals who have tumors in the cortical regions have a higher risk of seizure than those who have subcortical tumors^1–3,10^. This explains why patients with oligodendrogliomas which often locates in the white matter are more likely to experience seizures than those with astrocytomas.

In our study, brain tumors in the frontotemporal region have a higher rate of seizure activity than tumors located in another area, which is consistent with other studies’ result^2,3^. According to Engel, the mesial temporal lobe's intrinsic epileptogenicity contributes to the development of seizures^11^. Other authors suppose the hippocampus and amygdala are particularly known to be highly susceptible to epileptic activity. It contains a large number of excitatory neurons that are tightly interconnected, forming a complex network of neural circuits. Tumors that produce a mass effect and ischemia on surrounding or disruptions to this network can result in abnormal electrical activity that can trigger seizures^9^.

We noted that peritumoral edema is associated with an increased incidence of preoperative seizures. The similarity was found in other observations, peritumoral edema is a major epileptogenic factor in brain tumors^12–14^. The mechanism involves the mechanism of cerebral edema, which is caused by the association between vasomotor and cytotoxic disturbances. Intrinsic tumor hypoxia produces metabolic acidosis in the interstitial tissue and increases vascular endothelial permeability. The necrosis of tumor cells results in edema due to the disturbance of cell membrane permeability. These changes disturb the neurotransmitter pathways, thus creating epileptogenic characteristics. Therefore, the frequency of seizures appears to be higher in the tumor types with a significant degree of peritumoral edema^15^.

We did not find a statistical relationship between tumor size and epileptic rate. This could be explained by a wide deviation in the distribution of tumor size in our study. However, many authors agree that the larger the tumor size, the more correlated with the likelihood of epilepsy. Large tumors are more likely to cause compression, mass effect, and associated disorders at the cellular level. For meningiomas, Chen suggested that tumor diameters larger than 3 cm are more likely to induce seizures than small tumors^16^.

We demonstrate a high rate of seizure-free (Engel Class I) after gliomas (55–86%) and meningiomas (100%) removal surgery. Other studies have also reported a seizure-free rate ranging from 40 to 75% for glioblastoma, which is higher than in meningioma (60–80%) and glioneuronal tumors (70–90%)^6,8^. However, the treatment result not only varies between tumor types but also depends on the tumor location, the extent of tumor removal, and the manifestations of the previous seizure. Since a prolonged history of epilepsy was associated with a insufficient seizure control, it is reasonable to perform early surgical treatment for brain tumor-related tumor. Early surgery was also supported by other studies^17–19^.

LGG showed a lower control rate than HGG in our study. Interestingly, the Ki67 proliferation index was in a positive correlation with the seizure rate in LGG group. A higher Ki67 labeling index predicts a higher probability of uncontrolled seizure pre- and postoperatively. This is also demonstrated in Yuan’s study on grade II gliomas (OR: 4.354)^20^. In contrast, considering HGG-related epilepsy, the incidence of epilepsy in the high proliferation of Ki67 tumors was slightly lower than tumors with less Ki67 level. In a larger study investigating anaplastic gliomas (WHO grade III), Yang reported a similar correlation between seizure and Ki67^21^. However, a larger sample size and prolonged follow-up time are required to draw a more reliable conclusion.

Surgery for brain tumors aims to eliminate seizures and reduce the tumoral burden. We noticed that including the peritumoral epileptogenic foci in the gross-total tumor excision or extending the excision to the max safe limit substantially enhances survival rates in low-grade gliomas. This is why some authors recommend that resection should be adjusted to functional rather than oncologic limitations^22,23^. When feasible and safe, gross total resection is recommended to maximize the likelihood of epilepsy and oncologic control. This is especially true in cases with intractable epilepsy, or in those with low-grade tumors, who may achieve a survival time of decades^23^. However, unlike LGG, HGG usually requires radiation and adjuvant chemotherapy following tumor resection to improve seizure control^6^. Some publications support performing anterior temporal corticectomy and amygdalohippocampectomy in addition to gross total lesionectomy of tumors involving the temporal lobe to get a better result in seizure control (87–93% vs. 43–79%)^24–26^.

Until now, there has not been strong evidence of antiepileptic prophylaxis for patients who experienced preoperative epilepsy and underwent tumor removal. Following general practice, we continue antiepileptic drugs for 6 months after surgery and consider reducing the dose during clinical monitoring. The process of adjusting antiepileptic drugs is carefully considered, especially in cases with high-risk factors such as tumor genetic marker (e.g. IDH1 mutant), tumor location (e.g. frontotemporal lobe), the occurrence of interictal epileptiform discharges on EEG, and tumor residual after surgery. Patients can be gradually weaned from the ASM only if they witness no seizure afterward.

There has been insufficient evidence to support which specific ASM is more effective than others. Nevertheless, levetiracetam and valproic acid are the evidence-based first-choice^4,27,28^.

Our practice has generally chosen to apply either levetiracetam or valproic acid as the preferred anticonvulsant and combine both medications if the patient requires a higher step. Other ASM that has been shown effective and supported as mono-therapies and add-on agents are lacosamide, lamotrigine, and zonisamide^29,30^. If the monotherapy ASM shows inadequate seizure control, we usually add another agent as the supplementation rather than switching to the other monotherapies. 33.3% of patients achieved ASM-free at 12 months of follow-up in our study. Another study reported 2-year and 5-year ASM withdrawal rates at 42.9% and 72% respectively. A longer follow-up time (#3 years) may provide a larger rate of ASM withdrawal. ASM withdrawal contributed to improvement in neurological functions. The best time of ASM withdrawal for glioma remained debatable. Usually, ASM withdrawal was recommended after at least 1 to 2 years of seizure-free. Early ASM withdrawal had a significant risk for seizure recurrence. However, it did not influence the final seizure outcome, and most patients with seizure recurrence after ASM withdrawal could regain seizure-free after the administration of ASM.

Electrocorticography (ECoG) can detect precisely the position of the epileptogenic foci, and the ictal spread routes and help to optimize the resection under intraoperative guidance. ECoG has not been used consistently in this series of patients at our facility. Therefore, no concrete conclusion on ECoG application can be drawn from this study. The use of ECoG has largely depended on the surgeon's preference. ECoG was used more frequently in patients who had suffered from retractable epilepsy for a long time, especially when the tumor was stable and seizures were the more critical issue. There has been increasing in the number of evidence that supports using ECoG-guided guided surgery resection. Most studies suggest routine use of ECoG for those with a long history of uncontrolled seizures^23,31,32^. However, Englot believes the use of intraoperative ECoG during low-grade glioma and glioneuronal tumor resections was not associated with statistically different seizure outcomes, according to systematic studies of these procedures^18,33^. The role of ECoG in epilepsy-related tumor surgery still requires confirmation through large-scale randomized control studies to develop the best surgical approaches.

Despite providing meaningful results, our study has several limitations. Firstly, this was non-randomized study so selection bias may had influence on the outcome. Secondly, the number of patients enrolled to the study was relatively small, especially in some subgroups, which may influence the analysis result. Thirdly, the tumoral removal extent was heterogeneous, not only according to the presurgical evaluation but also referred to the ECoG, detailed deduction was hard to perform to obtain a powerful conclusion. Nevertheless, we hope our study provides valuable information on one of the most debatable topics in epilepsy surgery in the country and throughout South East Asia.

In summary, epilepsy is a disease burden that significantly affects the quality of life, particularly for those with brain tumors. From our study, surgical excision is a practical solution for reducing seizure occurrence. The likelihood of poor postoperative control increases with prolonged uncontrolled preoperative epilepsy and simple partial seizure type. In the long-term post-surgery, a seizure is more challenging to control in frontotemporal tumors, low-grade gliomas that have increased Ki67 profileration index or tumor that cause major peritumoral edema. Gross-total resection and early surgical treatment appear to be crucial factors that promote epilepsy control.

## Methods

A hospital-based prospective study was conducted at University Medical Center, Ho Chi Minh City, Vietnam between January 2020 and March 2022. The included patients underwent surgery for brain tumor-related epilepsy.

### Presurgical evaluation

In all patients, seizure was the first and only manifestation. Detailed epilepsy history and manifestation were documented. Data used for the analysis included the following clinical and demographic parameters: age of seizure onset, duration of epilepsy, age of surgery, seizure type, seizure frequency, and anti-seizure medications. Drug resistance was defined as failure to long-term (at least 1 year) adequate trials of two or more first-line ASMs. As for seizure frequency, patients with only 1 or 2 seizure attacks were categorized as a sporadic group. For patients with sporadic seizures or annual seizures, 24-h video electroencephalography (VEEG) (580-G2CGS S32, Biologic or EEG-1200C, Nihon Kohden) was advised. Ictal capture was considered usually in patients with more frequent seizures or drug-resistant epilepsy. All patients underwent 1.5-T (Siemens, Germany) or 3-T MRI (Siemens, Germany) scans. MRI parameters included tumor laterality, location and anatomic structures involvement, tumor volume, and the level of peritumoral edema on an imaging study, according to Schoenegger classification (major ≥ 1 cm, and minor < 1 cm)^34^. Epilepsy data were discussed in an epilepsy surgery conference with epileptologists, epilepsy surgeons, and neuroradiologists to determine the status and surgical candidacy of the patient. Moreover, final diagnosis, surgical procedures, and pathology were recorded as the primary outcome. All tumor cases enrolled in this study were reviewed and graded independently by a neuropathologist according to the 2016 World Health Organization (WHO) classification.

### Surgical strategy

ECoG (NicoletOne, USA) use was not randomized, which was determined by the appointment of an epileptic electrophysiologist and the economic burden of patients. In most patients who underwent ECoG, the surgery was assisted by the ECoG. Neuroleptanalgesia (fentanyl) has been the traditional method of sedation because neither drug affects intraoperatively recorded interictal epileptiform activities (IEAs). To facilitate ECoG recordings, anesthesia must be minimized or eliminated, with decreased levels of intravenous and/or volatile agents. The use of muscle relaxants at the time of ECoG will also help to ensure that the patient does not move. Volatile anesthetics can modulate neuro excitability in a dose-dependent manner, manifested most prominently at near burst-suppression doses (1.5 minimum alveolar concentration [MAC]) and being minimal or absent at low doses (0.3 MAC). Because Dexmedetomidine (DEX) (which is suggested to have advantages over Propofol in general anesthesia) is not yet available in Vietnam, Propofol-based general anesthesia was administered instead. DEX is especially used in awake craniotomy because it appears to be associated with less neurocognitive impairment. Intraoperative seizure detection rates were higher with DEX than with Propofol. After 30-min stop of Propofol, a 4-contact or 8-contact strip (Ad-Tech Medical, USA) was placed on the surface tumor area. When single spikes, polyspikes, or rhythmic spikes were found, extended resection was performed. Lesionectomy was defined as surrounding brain tissue removal less than 0.5 cm. The extent of tumor removal (subtotal, gross total, extra-lesional resection) is classified according to postoperative CT scans and surgery reports. Extra-lesional resection is removal that extends beyond the tumor margin or signals abnormality on imaging study. Postoperative information includes histopathology, surgery complications, tumor recurrence on MRI, and epileptic activity on EEG at follow-ups. The Engel Classification of Seizures was used to assess epilepsy outcome postoperatively, at 6 and 12 months following surgery: Class I, seizure free; Class II, rare seizures; Class III, meaningful seizure improvement; and Class IV, no seizure improvement or worsening). KPS score was used to measure the secondary outcome of the patients.

### Statistical analysis

We carried out analysis addressing the relation between demographic and tumor removal-related variables, perioperative seizure control, and seizure condition at 6 and 12 months follow-up. The Chi-square test and Fisher exact test for dichotomous variables, and logistic regression for continuous variables were used in univariate analysis. Epilepsy outcome was dichotomized as seizure free (Engel Class I) versus no seizure free (Engel Class II–IV). Variables from univariate analyses with probability values < 0.05 were added to a backward stepwise logistic multivariate regression.

### Ethics considerations

All procedures performed in studies involving human participants were in accordance with the ethical standards of the institutional and/or national research committee and with the 1964 Helsinki Declaration and its later amendments or comparable ethical standards. The study was approved by the Bioethics Committee of the University of Medicine and Pharmacy, Ho Chi Minh City (No: 631/HĐĐĐ). The informed consent form contains extensive information about this trial, including the purpose of the trial, the study design, potential risks and benefits associated with the trial, and the participant’s rights and responsibilities. All participants are informed that the data collected is for research purposes only, not for any other purpose and all information collected is kept confidential and encrypted. Informed consent was obtained from all participants and/or their legal guardians.

## Data Availability

All data generated or analysed during this study are included in this published article. All authors reviewed the manuscript.
